# Reconsidering editorial consideration: Changing editorial assessment could reduce subjectivity in the publication process

**DOI:** 10.15252/embr.202358127

**Published:** 2023-09-25

**Authors:** Shai Berlin

**Affiliations:** ^1^ Rappaport Faculty of Medicine Technion Haifa Israel

**Keywords:** Science Policy & Publishing

## Abstract

Can rethinking editorial assessment reduce subjectivity and transform the way science is communicated?

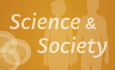

What defines good science? While subject to debate, one definition that most scientists would agree on is that *good* science follows the fundamental guidelines of the scientific method, namely the formulation and testing of hypotheses based on experimental observations and inferences. Importantly, *good* science should be defined by objective criteria and be amenable to scrutiny by “testable and refutable” means (Thornton, 2022). It is safe to assume that most scientific journals' endeavour is to publish science of high quality—that is, *good* science—judging by the time, efforts and money invested in the evaluation and publication of manuscripts. These include pre‐review editorial assessment, formal peer review and a final editorial judgement. However, while reviewers judge the soundness of the science, editors appear to act as “gatekeepers”, who determine what is relevant science for the journal (Dance, 2023).

Editorial assessment of relevancy, in particular at the pre‐review stage, includes evaluations of journal‐specific criteria: such as whether the work falls within the journal's scope, adherence to technical guidelines, quality of the language and so on. But it also includes evaluation of whether the science is of interest to the journal. Indeed, a quick survey of various top biomedical journals' guidelines—the “Aims and Scope” section to inform authors of the journal's selection criteria—disclose that these aim to publish reports that are impactful, insightful, timely, elegant or “of surprising conclusions”, to name a few (www.nature.com/nature/journal‐information). These vague terms are all subjective and thereby open to different interpretations by different individuals.

Subjectivity has a role in science. It helps authors to evaluate their work and choose the appropriate channels and routes for communicating it. For editors, it helps filter submissions, provide focus or highlight trends in science, and so on. Such subjective considerations may increase interest within or outside academia and thereby directly benefit authors by exposing their work to a potentially wider audience. Subjectivity also helps editors select manuscripts that may fare well in the review process to shorten the time to publication, reduce reviewer workload and increase acceptance rates (Laursen, 2021). Nonetheless, subjectivity has its negative sides, as subjective criteria can be differently interpreted by different individuals, cannot be standardized between manuscripts or journals and may be skewed by personal preferences and views, journal‐specific criteria and economic pressures, academic backgrounds, inherent biases and so on. The main question is what is the extent of subjectivity in editorial assessment?

## Pre‐review editorial consideration is primarily subjective

The aim of the pre‐review consideration by a scientific editor is to select submissions for review as opposed to first submitted and first reviewed. This step is important to protect reviewers from wasting their time with unsuitable or irrelevant manuscripts, especially with the ever‐growing number of submissions. However, the pre‐review editorial consideration is responsible for the vast majority of rejections, up to 90% in some journals (Laursen, 2021), which cannot simply be explained by lack of suitability, lack of adherence to journal guidelines or other objective criteria. Furthermore, pre‐review editorial assessment usually does not consider inappropriate methodology, inaccurate conclusions or poor or incomplete analysis as these are typically handled by experts during the review stage.

Thus, it strongly implies that most initial rejections are based on subjective criteria that come on top of and overrule objective measures. Notably, there is very little literature that provides details about the reasons for pre‐review editorial rejections, which goes against the current push towards transparency in scientific publishing (Siler *et al*, 2015). Even when authors are provided with a “detailed” rejection letter, it mostly includes vague and generic arguments that the paper is of insufficient novelty or not relevant enough, without further explanations as to why or for whom, lending further support that this step does not follow objective decisions based on well‐defined criteria.

Notably, there is very little – or even a complete lack of – literature that presents statistics and details regarding the reasons for pre‐review editorial rejections…

Take, for instance, the issue of novelty. Novelty may cover research that introduces a new field of study or that describes a discovery of a new organism, cell type or disease. However, novelty can also be found in smaller details, such as discovery of previously unreported mutations that instigate a known disease. Whereas the mutations are novel, it can be argued that the extent of novelty is moderate owing to their similarity with other previously described mutations, and so forth. Unfortunately, there is no reliable method to quantify the “extent of novelty” and there is no educated or scientific method for authors to align their understanding and their appraisal of these variables with those of the editors. Moreover, negative or confirming data can also be said to be novel by disproving a popular hypothesis, for instance, or by providing additional evidence to support earlier published work. Nonetheless, either case would likely be much harder to get accepted for publication, considering the rather narrow concept of “novelty” that is used by many journals.

Importantly, neither do editors have scientifically sound methods for evaluating other subjective criteria, such as impact, expected size of readership and timeliness—not to mention whether a paper is boring (this is not a typo: Thrower, 2012). In fact, it is repeatedly shown that attempts to predict the impact of a publication fail to yield reliable outcomes (preprint: Lariviere *et al*, 2013). Anecdotally, some of the breakthrough publications that eventually were awarded the Nobel Prize were declined by scientific editors at top journals (Siler *et al*, 2015). A recent example was the editorial rejection of Virginius Šikšnys by *Cell*: his paper was the very first that described the application of the bacterial CRISPR/Cas system for genome editing.

Importantly, neither do editors have scientifically‐sound methods for evaluating other subjective criteria such as impact, expected size of readership, timeliness, and so on.

## Centralization of responsibilities

The role of editors does not end there. In effect, the entire process is centred around editors, which concentrates all duties—judge, jury and executioner—on a single person, or a small group of people. In general, editors play a crucial role in ensuring the quality and integrity of the peer review process by selecting reviewers, evaluating their comments and recommendations, clarifying any conflicting comments, intervening if necessary or requesting additional reviews if needed. However, editor intervention in reviewers' comments is often not transparent or standardized as each manuscript is treated independently and differently, thereby making the process inconsistent across different submissions.

In effect, the entire process is centred around editors, which concentrates all duties onto a single person, or a small group of people.

Finally, editors also have the final say on whether to accept or reject the paper. At this step, their considerations are certainly more objective, as they are now equipped with evaluations of the *goodness* of the science from reviewers. However, post‐review assessments may still include considerations for novelty, relevance or size of potential readership should the editors find these relevant for the journal's agenda.

## Rejection rates are detrimental to science

The high rejection rate is disruptive to science and, on average, the publication process is getting slower and longer (Dance, 2023). It is obvious that this has negative effects on authors' motivation and slows down the dissemination of results. It also fuels the already extreme competition in academic research that compels authors to publish in a few high‐impact journals with even higher rejection rates—and likely even more reliant on subjective criteria.

Another negative outcome of research is the dimming effect. This term is used here to describe a scenario whereby less popular fields of research find it more difficult to get published in top‐tier biomedical journals because the topic is perceived as less “impactful”, “important” or any other subjective terms or that would not attract enough readers (see recommended reading list). Less chances for publication in highly selective journals may translate to lower chances to get funding; reducing the ability of research groups to maintain their output. For example, the US National Science Foundation (NSF) uses a merit‐based system to determine which research proposals will receive funding. Proposals that are deemed to have the greatest potential for scientific or technological advancement judged by publications are given priority. Even more troubling is the effect on the reputation of the research group, which can have a substantial negative effect on hiring. As graduate students and postdocs are facing ever‐increasing competition over academic and industrial positions, it is therefore not unexpected that they may prefer research groups with a higher probability of publishing in top‐tier journals. This further reduces the pool of good candidates who are interested in certain research areas and thereby enhances the dimming effect.

Collectively, these considerations call into question whether the primarily “subjective pre‐review assessment” has a meaningful role in science. There is no question that a certain filter is required, including quality checks, otherwise, reviewers will be swamped with work—as is, review acceptance rates have been dropping for years (Dance, 2023). However, filtering should be based on quantitative means, and not on subjectivity. Even if editors and editorial boards do their best to stay clear of some or perhaps even most subjective criteria, the extensive rejection rates and the non‐transparent nature of editorial decisions at many journals make it very hard to address and remedy the situation.

## Editorial pre‐review is obsolete

The pre‐review editorial consideration is not only subjective and detrimental to science but it is also obsolete. On a technical level, many “mundane” chores at the pre‐review stage could be automated or done by technical experts, such as checks for completeness, plagiarism, image manipulations and language quality. Interestingly, different journals employ quality checks at the pre‐review stage to varying degrees (or not at all). Perhaps, it is time to standardize this step. This should further weed out unsuitable manuscripts, which both editors and reviewers are likely to benefit from. Moreover, this streamlining should help to reduce the time between submission and publication.

Other technical tasks that may require human attention, for instance, communicating with authors, can be tasked to technical experts instead of scientific editors. In the case of professional editors, this would probably reflect the same person; however, the editor, aka technical expert, would no longer be required to make decisions based on subjective criteria, but would only need to decide if the work in question fulfils a set of well‐defined criteria before it is sent to peer review. These criteria could be established by the journal or by researchers themselves, and if posted online, would better serve authors to decide whether their reports meet these requirements as opposed to the current “Aims and Scope”. This would also entail abandoning ill‐defined criteria, such as novelty or timeliness, and other non‐quantitative measures that have not been specifically defined *a priori*. The same expert would then draft the final decision solely based on external reviews. Lastly, editorial consideration is somewhat redundant, as several of their roles overlap with those of the reviewers, rendering it inefficient. Scientific journals could therefore profit twofold by eliminating the pre‐review consideration: save money on salaries and shorten time to publication.

## Abandoning editorial pre‐review

Abandoning the subjective pre‐review editorial assessment may appear revolutionary and controversial, however, there are signs that things are already heading in that direction. For instance, *PLoS One* was among the first journals to forego subjective criteria such as novelty or relevance. *Review Commons* and *Peer Community In* offer journal‐independent review of pre‐print manuscripts without journal‐specific criteria, although they do maintain the authority to editorially reject papers. The editorial decisions are intended to be suitable for a collection of different journals, likely rendering their definitions of novelty, interest or impact broader or more relaxed than in standard journals. After review, the authors may choose to transfer their manuscript alongside reviews and revisions to different journals within the initiative. Once there, however, the manuscript remains bound to journal‐specific criteria and subjectivity.

Another attempt to bypass subjectivity has recently been introduced by *eLife*. Their latest policy empowers authors to decide whether or not to publish their manuscript in the journal irrespective of the reviewers' reports, thereby circumventing the final editorial decision. However, it does not resolve the underlying problem of pre‐review editorial subjectivity; instead, it worsens it by putting more emphasis on the pre‐review step and therefore even more power in the hands of editors and the pre‐review editorial consideration step.

A potential solution is a system without editors and without pre‐review evaluation. While the advantages are obvious for the scientific community, it must be appreciated that adopting an editorial‐free system would entirely change the way science is communicated and evaluated. First, an editorial‐free system would likely drown scientific journals in submissions, especially top‐tier journals where many scientists strive to publish. Unless provided with added incentives, such as financial compensation, acceptance rates by reviewers are likely to plummet further, exacerbate delays in publication and reduce the quality of reviews.

These problems may motivate authors to seek alternative publication routes, such as pre‐print servers. Naturally, many scientists may be deterred from doing so, as pre‐prints are not peer reviewed and researchers may fear that the community would not seriously consider their manuscript and results, especially in the biomedical fields. However, pre‐prints are becoming increasingly accepted by the scientific community, not to mention major publishers, and are taken quite seriously, as attested by the growing number of cited pre‐prints.

## The advantages of pre‐prints

Pre‐prints offer multiple benefits over journal‐based publications by immediately exposing the science to a larger, more diverse audience, offer *scooping* protection, are citable and can still be submitted to traditional journals. Importantly, pre‐prints are curated to a certain degree for completeness, quality, language and so forth, minimizing posting of subpar reports. The one major setback with this system is the poor engagement of the research community—so far only 5% of pre‐prints have received comments although gradual increases are noted (Brainard, 2022). However, this scenario will necessarily change when the ball starts rolling.

If scientists gradually abandon the traditional journal route to publication, it may also demotivate them from reviewing for traditional journals. This may engender a snowball effect whereby more and more authors revert towards posting pre‐prints, coinciding with less and less acceptances to review for journals. Scientists already dedicate a substantial amount of their time towards reading manuscripts including pre‐prints for their personal education and research, thus abandoning reviewing for journals would mean that they will have more time for commenting and reviewing the pre‐prints they read anyway. They will not only be able to do so at their own pace but they should also have the freedom to decide what they find worthy to review instead of being handed reports after editorial selection. Additional means to increase engagement may include reviews based on journal clubs or group seminars, in particular when it turns out to be enjoyable and educational to read, comment on and discuss manuscripts (Richter *et al*, 2023). This could be incorporated as a mandatory graduate course.

To further boost online engagement, scientists may also be incentivized to review pre‐prints. Recent reports suggest that scientists are more motivated to review if they are credited for it, especially if these efforts are recognized by their employers; monetary compensation ranked only sixth (Hayes & Hardcastle (2019))(see recommended reading list). This is not far‐fetched as many academic institutions are already incentivizing a variety of other activities to increase and ensure engagement of faculty members, such as compensations for reviewing PhD theses, for obtaining external grants and a variety of other rewards and prizes. Likewise, funding agencies can request grant holders to perform a minimal number of online reviews as part of their deliverables. Indeed, major funding agencies, such as the ERC, already encourage dissemination of science funded by them by any means as long as it is not buried behind paywalls, so why not explicitly request publications and reviews of pre‐prints?

…as can be learned from the COVID pandemic, scientists are able to quickly adapt to new communication routes.

Additional factors to promote reviewing and commenting on pre‐prints may include updating scientists about new pre‐prints in their field, online group discussions, allowing scientists to post opposing results alongside the pre‐print and so on. Obviously, this will take time to implement but, as can be learned from the COVID pandemic, scientists can quickly adapt to new communication routes (Brainard, 2022).

## The downsides of pre‐prints

Nonetheless, there are downsides to pre‐prints. One example is the posting of dubious or fraudulent publications which are challenging to detect, even in the traditional journal‐based publication route (Cyranoski, 2014). Even if editors and reviewers do weed out erroneous and fraudulent papers, there are many instances where erroneous and fraudulent articles were identified by the community after reviewed publications (Williams *et al*, 2019). These cases not only exemplify the self‐correcting nature of science but also highlight the need for time to allow the community to analyse the data and its interpretation. This has proved to be the case with superluminal neutrinos, primordial gravitational waves (Cowen, 2015) or the link between cell phone radiation and cancer (preprint: Wyde *et al*, 2016).

On a similar note, how can erroneous, irrelevant or unsuitable comments be prevented? One means is by requiring author identification, such as ORCID. Moreover, comments could be curated for content to prevent harmful or offensive remarks. Of note, these issues have not been a real concern so far.

A related concern is comments by non‐experts. This somewhat equates to challenges in traditional journal‐based reviews when selected referees are experts in some but not all aspects of the paper (see recommended reading list). In fact, there is no clear definition of what constitutes an expert reviewer, and the criteria for selecting reviewers may vary across disciplines, journals and editors. These could be easily remedied if the referees were obligated to identify themselves and disclose which part of the paper they reviewed. Regardless of whether the comments were provided by experts or not, authors should be able to respond and address irrelevant comments.

## Slow change

Unfortunately, many scientists still prefer to submit to top journals given the irresistible lure of high‐impact factor journals and the notion that publications therein greatly increase a researcher's chance to get funding, prizes or to secure a position in academic research. Second, there is the widespread assumption—especially in biomedical fields—that only scientific journals can provide the “seal of approval” for scientific integrity and validity. Thus, it appears that the greatest problem is the scientific community itself and its concerns. While pre‐prints or other means provide a viable solution to the *subjectivity* problem, it also requires funders and institutions to adopt the DORA principles, and to judge scientists' contributions solely by what is described in their publications and not based on where these were published. This should empower scientists and relieve their concerns to some extent. Ultimately, for this change to ensue, more scientists need to change their habits and start appreciating their colleagues' work irrespective of whether this was published in high‐impact journals or not.

One means to drive this change is by convincing institutions and agencies to mandate posting of pre‐prints. First, pre‐prints bear no cost to authors, which is in line with the current upheaval surrounding the high publication fees charged by many journals (see recommended reading list). Second, it is free and accessible to anyone, which increases the visibility and impact of the research by reaching a wider audience. Third, it is faster, both in terms of dissemination and the progression of science as a whole. It thus allows students to finish their degrees on time and to establish credit and priority. Moreover, pre‐prints can facilitate collaboration among researchers as they can invite comments, suggestions and corrections from the scientific community. Finally, it levels the field between different countries, institutions, fields of study, different positions of researchers and author seniority.

## Changing the system one editor at a time

Understandably, there are many concerns about a scenario that abandons submissions to scientific journals at large. Who will validate publications and assess whether the results are sound and correctly interpreted? How should funding agencies decide whom to fund? How will institutions assess the quality of applicants for open positions, promotions and so on? I believe the answer is quite simple: evaluate the science, rather than rely on the judgements and pre‐selection of editors, which is becoming the norm in several areas, such as academic promotions.

But can we trust scientists to assess the quality of their colleagues' work impartially? Universities, institutions, funding agencies and journals certainly do. Scientists conduct research, educate the next generation of scientists, review grant proposals and so on, all of which are based on the notion that scientists act professionally and fairly. Moreover, scientists are trusted by funding agencies to efficiently manage the money they obtain to carry out research. Thus, why should not others do so? Given the right environment, with reduced publishing pressures and less competition over select journals, we could rid ourselves of the perpetuated impact factor madness and the toxic environment it has created.

Removing editorial consideration from the publication process is not a panacea for all problems that plague science and publishing. But it would at least greatly reduce the subjectivity in the publication process and emancipate scientists from perpetual submissions–rejections rounds and from tiresome and lengthy review duties to assess if a paper is sufficiently “novel” and “relevant”. It would free scientists' time to read and comment on pre‐prints instead and make scientific research and findings accessible by anyone. This could engender a new culture of online commenting and reviewing and pave the way for new means of communication and interaction between scientists. For instance, posted manuscripts could be progressively modified or corrected in response to online comments, or other scientists could even post their own results alongside another pre‐print in support or opposition to the results presented and their interpretation. This creates a scenario where smaller pieces of data—not enough to justify a full paper—can supplement other manuscripts, which actually reflects the “scientific endeavour” by allowing individual scientists to directly contribute to the greater task of understanding the world.

### Disclosure and competing interests statement

The author declares that he has no conflict of interest.

### Recommended reading list

#### Journal publication fees

CBC News (2015) Academic publishers reap huge profits as libraries go broke | CBC News.

Fazackerley A (2023) ‘Too greedy’: mass walkout at global science journal over ‘unethical’ fees. *The Observer*.

Hagve M (2020) The money behind academic publishing. *Tidsskr Den Nor Legeforening* https://doi.org/10.4045/tidsskr.20.0118

#### Methodological quality of scientific experiments and rank of the journal

Brembs B (2018) Prestigious science journals struggle to reach even average reliability. *Front Hum Neurosci* 12

#### Dimming effect

Anon (2021) The social sciences are useless. So why do we study them? Here's a good reason: | Statistical Modeling, Causal Inference, and Social Science https://statmodeling.stat.columbia.edu/2021/03/12/the‐social‐sciences‐are‐useless‐so‐why‐do‐we‐study‐them‐heres‐a‐good‐reason/ (accessed 8.12.23).

#### Postdoc crisis

Woolston C (2020) Uncertain prospects for postdoctoral researchers. *Nature* 588: 181–184 https://doi.org/10.1038/d41586‐020‐03381‐3

#### Advantages of Preprints

Bourne PE, Polka JK, Vale RD, Kiley R (2017) Ten simple rules to consider regarding preprint submission. *PLoS Comput Biol* 13: e1005473 https://doi.org/10.1371/journal.pcbi.1005473

#### Reviewer selection

Tennant JP, Ross‐Hellauer T (2020) The limitations to our understanding of peer review. *Res Integr Peer Rev* 5: 6 https://doi.org/10.1186/s41073‐020‐00092‐1

## Supporting information


