# Supplementary material for: Reconsidering editorial consideration: Changing editorial assessment could reduce subjectivity in the publication process
Source: EMBO Rep. 2023 Sep 25;24(11):e58127. doi: 10.15252/embr.202358127 (PMC10626424; doi:10.15252/embr.202358127)
Supplement: Supplementary file 1 [file EMBR-24-e58127-s001.pdf]

EMBO reports

Reconsidering editorial consideration

Shai Berlin
DOI: 10.15252/embr.202358127

Corresponding author(s): Shai Berlin (shai.berlin@technion.ac.il)

Review Timeline:

Submission Date:	6th Sep 23
Accepted:	15th Sep 23

Editor: Holger Breithaupt

Transaction Report: This article was editorially reviewed.
